# A Further Investigation of the Effects of Extremely Low Frequency Magnetic Fields on Alkaline Phosphatase and Acetylcholinesterase

**DOI:** 10.1371/journal.pone.0148369

**Published:** 2016-03-10

**Authors:** Gary Silkstone, Michael T. Wilson

**Affiliations:** School of Biology, University of Essex, Wivenhoe Park, Colchester, Essex, CO4 3SQ, United Kingdom; National Research Council, ITALY

## Abstract

Using a custom build spectrophotometer equipped with Helmholtz coils and designed to study the effects of magnetic fields on enzyme reactions in real-time we have investigated the influence of fields, from 100 μT to 10 mT and at a variety of field frequencies, on the membrane bound enzymes alkaline phosphatase and acetylcholinesterase. We have also employed other methods to apply a magnetic field, e.g. Biostim. In contrast to earlier reports we have been unable to detect any field effects on these enzymes under any field/frequency regime. We discuss possible reasons for the discrepancy between this and earlier work and note the particularly complex influence of small temperature changes that may confound analysis.

## Introduction

Over many years, and especially more recently, there has been considerable interest in the effects of magnetic fields on biological systems [**1–10**]. Much of this interest has focused on the effects of low intensity fields on the reactivity of light induced radicals. The reason for this is that there is good experimental evidence that navigation during the migration of some birds and insects is partly based on sensing the earth’s magnetic field [**6–10**] and that photoactivated cryptochrome, a flavoprotein located in the eye of the organism, is the magnetosensor [**6, 11–13**]. Furthermore, there is a well established theory that can provide a mechanism through which low strength magnetic fields may influence the temporal behavior of correlated radical pairs [**6, 11**], such as may be formed in cryptochrome during photoreduction or, possibly, during re-oxidation of the photoreduced protein by oxygen [**14**].

Alternative radical based mechanisms through which biological systems may be influenced by magnetic fields have also been advanced. For example, Buchachenko and co-workers have proposed a mechanism based upon the fact that one of the isotopes of Mg, namely ^25^Mg, possesses a magnetic nucleus [**15**]. The proposal is that Mg participates in the mechanism of Mg dependent kinases through phosphate-to-Mg electron transfer forming a radical-ion-pair and that the two spin states of this pair, singlet and triplet, contribute differently to ATP synthesis with the triplet state being the more efficient. Because of coupling between the unpaired electron and the magnetic nucleus, ^25^Mg was proposed to enhance the activity of kinases by inducing a higher population of the triplet state and to make the enzyme highly sensitive to applied magnetic fields. Buchachenko *et al*. reported an increase in the rate of ATP synthesis catalysed by creatine kinase (*V*. *Xanthia* venom) in the presence ^25^Mg and an increase by 50% at ~50 mT field strength. Recent attempts to reproduce these results have, however, failed [**16**].

In 2005, Morelli *et al*. (2005) studied the effects of extremely low frequency (ELF) electromagnetic fields (EMF’s) on several (seven in total) membrane-associated enzymes [**17**]. They claimed that the activities of three of these (alkaline phosphatase, acetylchlinesterase, and phosphoglycerate kinase) exhibited a decrease in activity (about 50%) when exposed to ELF EMF’s and that this effect disappeared when the enzymes were dissolved in a buffer containing the detergent Triton x-100, suggesting that fields may modify the membrane organization and structure by acting directly on the anisotropy of diamagnetic susceptibility of membrane phospholipids. In subsequent studies on acetylcholinesterase activity in cerebellum synaptosomal membranes, Ravera *et al*. (2010) reported the effects of magnetic fields (50 Hertz sinusoidal) exhibiting a sharp threshold, having no effect below a certain field strength and a significant effect above this [**18**]. This behaviour is strongly reminiscent of a phase change in the membrane lipid and this is a possible mechanism through which magnetic field influence membrane bound enzymes. Previous physico-chemical studies by Caseli and co-workers support the findings of Morelli *et al*., where lipid-anchored alkaline phosphatase incorporated into artificial phospholipid monolayers showed a substantial decrease in enzyme activity following a phase transition on applied increases in pressure [**19**]. Indeed, observed magnetic field effects on the enzyme acetylcholinesterase are quite numerous [**18**, **19–21**], as they are for a few other membrane bound enzymes [**5**]. Most of these reports focus on the effects of magnetic fields on these enzymes being attributed to the diamagnetic anisotropic properties of the membrane phospholpids to which they are bound [**5**].

The intriguing findings of Morelli *et al*. have important implications for biology and indeed human health, and prompted us to further investigate the effects of magnetic fields on the activity of alkaline phosphatase (from a microsomal preparation from fresh bovine liver) and acetylcholinesterase (from ghost cell membranes from fresh erythrocytes). We have adhered rigidly to the protocols reported by Morelli *et al*. for enzyme preparation (ensuring that the enzymes remain membrane bound) and for the assay systems. In addition we have employed a specially designed spectrometer equipped with paired Helmholtz coils capable of real-time spectroscopic assay [**16**]. For consistency with the earlier work we have also applied magnetic fields using the Biostim equipment (see Materials and Methods), that was used by Morelli *et al*. The Biostim is commercially available and has been used clinically to aid bone healing following fracture and the findings that this device decreases the activity of specific membrane-associated enzymes may therefore have important implications for our understanding of the proposed beneficial effects of magnetic fields. With this in mind we consider it warranted to revisit these studies given their possible medical importance and the use of the Biostim in clinical contexts.

The real-time spectroscopic assay we employed has advantages over the fixed time point assay used by Morelli *et al*. *as* it enables one to follow entire time courses and to apply fields of a chosen strength and frequency continuously or in an “on and off” manner at any time during the time course of a reaction, thereby permitting direct observation of any changes in the reaction rate on application of a field.

## Materials and Methods

The blood samples used in this study were sourced from the blood bank based at Colchester General Hospital,Turner Road, Colchester, Essex, CO4 5JL, U.K. (URL www.colchesterhospital.nhs.uk/pathology). These samples were of anonymous origin and is standard procedure. These samples were of course suitably screened by the Hospital's Pathology/Haemotology Departmant for the presence of the main blood bourne diseases such as HIV, HBV, HCV, HTLV, Syphilis, Malaria, to name but a few, to make them safe and so available for patient donation. Our laboratory based at the University of Essex and where this work was conducted, is one that specialises in handling blood samples like those of donated blood obtained from Hospitals, as we work predominantly on the biochemical mechanisms of how haemoglobin functions.

### Magnetic field production

#### Dual Helmholtz coil/spectrometer system

We have constructed a dual beam spectrophotometer with sample and reference compartments each between paired Helmholtz coils. The coils surrounding the sample apply a field, static or at a selected frequency, while those of the reference are counter wound so that no field is produced but all heating and vibration effects are equivalent to the sample coils. The generator system supplies a sinusoidal wave shape on application of a desired frequency. The dual Helmholtz coils were placed approximately 1 m apart to avoid any mutual stray fields. Care was taken to ensure that no metal (especially iron) containing materials were in the local vicinity of the apparatus, and all the equipment was arranged on a wooden bench and away from electrical wires. These procedures ensured that no unwanted fields were detected in the vicinity of the apparatus on mapping the area using a Hall probe. The spectrophotometer can follow the activity of enzyme reactions in real time. The enzyme reaction mixture is split, one portion placed in each coil, and both samples are monitored. The apparatus has been fully described previously [**16**].

The temperature was maintained at a 25°C throughout all experiments and, most importantly, any temperature difference between the two reaction mixtures one where a field could be applied (sample) and the other not (reference) was kept below 0.1°C to avoid any confounding influence arising from temperature dependent activity differences between the two reaction mixtures. The apparatus was housed in a constant temperature room and the optical cuvettes placed in custom built Perspex water jackets connected to a temperature controlled water bath. The coils were equipped with individually shaped aluminium cooling fins to ensure that their temperatures were identical. In addition the temperature was monitored periodically using thermometers (accurate to 0.1°C) placed in the two reaction mixture cells, sample and reference. The specific temperature of 25°C that we used was identical to that reported by Morelli *et al*. for their studies.

#### Biostim system

ELF EMF’s were also produced by an apparatus (Biostim Igea, Modena, Italy) used mainly for clinical applications (e.g. accelerate the healing of bone fractures). The generator system supplies a square wave with a maximal applied tension of 180 V, a period of 13.3 ms (75 Hz of frequency), a duty cycle of 10%, to a couple of Helmoltz coils (each with 1000 turns of copper wire of 0.2 mm of diameter) with internal and external diameter of 72.5 and 82.5 mm, respectively. Measurements of the maximal intensity of the magnetic field with a gaussmeter, showed that it was fairly constant midway between the coils, giving values of about 2.5 mT at a distance of 3 cm.

### Sample preparations

#### Microsome preparation

Fresh (~1 hour *post mortem*) bovine liver was chopped and homogenized (Waring/Commercial Heavy Duty Blender) in Tris–HCl (20 mM, pH 7.5) containing 0.25 M sucrose. The homogenate was centrifuged for 10 minutes at 1000g. The supernatant was collected and centrifuged for 20 min at 20,000g. The resulting supernatant containing cytosol and microsomes was collected and centrifuged at 100,000g for 1 hour. The pellet containing microsomes was stored at -80°C.

#### Ghost cell membrane preparation from human erythrocytes

Starting from 20 ml human blood treated with 1 mg/ml EDTA (to avoid coagulation). The sample was centrifuged at 3000g for 15 minutes at 4°C and the pellet containing erythrocytes was collected and re-suspended in 150 mM KCl and 20 mM Tris–HCl, pH 7.4 and centrifuged/washed three to five times depending at 5000g for 15 minutes. To obtain the erythrocyte membranes, the last pellet was re-suspended in hemolysis buffer containing 1 mM EDTA and 10 mM Tris–HCl, pH 7.4 for… and then centrifuged at 20,000g for 30 minutes. The pellet containing erythrocyte membranes was re-suspended in a few milliliters of distilled water. Samples were stored at -80°C.

### Enzyme activity measurements

#### Alkaline phosphatase activity

Alkaline phosphatase activity was measured by using 4-nitrophenylphosphate as a substrate and monitoring 4-nitrophenol formation. The substrate concentration range studied was from 0.07 to 5 mM. Microsomes from liver were added to a reaction mixture containing 4-nitrophenylphosphate, 0.1 M glycine buffer (pH 10.5), 0.1 mM ZnCl_2_, and 1 mM MgCl_2_. The increase in absorbance at 405 nm (ε = 1.85 mmol^-1^ cm^-1^) is proportional to the nitrophenol produced. Microsome concentrations were varied in order to optimize reaction times.

#### Acetylcholinesterase activity

Acetylcholinesterase activity was measured by using acetylthiocholine chloride as substrate and monitoring the thiocoline production. The substrate concentration range studied was from 0.025 to 0.40 mM. Ghost erythrocyte membrane preparations were added to a reaction mixture containing acetylthiocholine in 50 mM phosphate buffer, pH 7.2. DTNB (0.5 mM) was added. Thiocoline reaction with DTNB formed thionitrobenzoate (ε_405_ = 1.33 mM^-1^ cm^-1^) which was monitored spectrophotometrically by following the rise in absorbance at 405 nm. Ghost erythrocyte membrane concentrations were varied in order to optimize reaction times.

#### Determination of protein concentrations

The activities of the enzymes alkaline phosphatase and acetylcholinesterase in the work of Morelli *et al*. were expressed in U/mg (defined as n/moles of substrate transformed/min/mg protein). Therefore, the total amount of protein in our enzyme samples where microsomes (for alkaline phosphatase) and ghost erythrocyte membranes (for acetylcholinesterse) were being used was calculated. This was achieved by carrying out Biuret (protein determination) assays using known amounts of microsomal and ghost erythrocyte membrane samples [**22**]. A standard curve was made by adding known amounts of bovine serum albumin (BSA) (10 mg/ml stock) to Biuret reagent, the concentration range of BSA being 0–0.5 mg/ml final in 0.1 mg/ml increments, and leaving to stand for 15 mins at room temperature. An increase in absorbance (540–460 nm) was observed on increasing [BSA], and a line of best fit plotted to the data points. Suitable known dilutions of microsomes and ghost erythrocyte membranes were made such that following the Biuret reaction as described for the standards, the final absorbance value obtained was within the range of the standard curve. All experiments were carried out in duplicate.

### Outline of MFE exposures we and Morelli’s group used

To fully understand the MFE exposure conditions/systems we and Morelli’s group (2005) used in the studies on both enzymes alkaline phosphatase and acetylcholinesterase, these are set out in Table 1.

**Table 1:** Different MF conditions are denoted as numbers 1, 2 and 3. T = tested and NT = not tested.

MF conditions	Biostim	Dual Helmholtz/ spectrophotometer
**1** 2.5 mT, 75 Hz, square wave, 10% on/off duty cycle	***T Morelli and Silkstone***	***NT***
**2** 2.5 mT, 75 Hz, sinusoidal wave	***NT***	***T Silkstone***
**3** 2.5 mT static field	***NT***	***T Silkstone***

## Results

**Fig 1:**
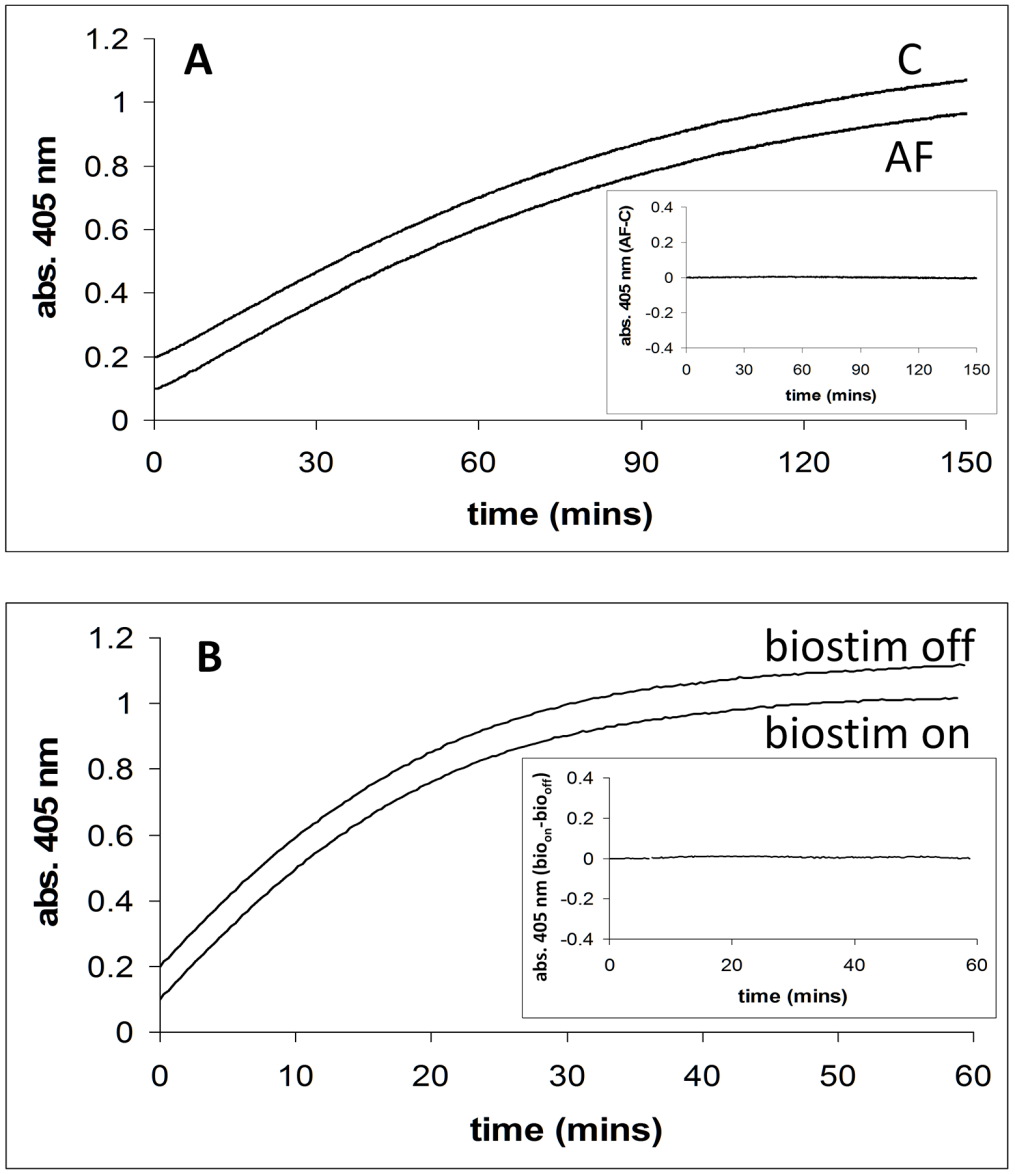
Alkaline phosphatase activity in the presence and absence of applied magnetic fields. **A**. Alkaline phosphatase activity in the presence (2.5 mT/75 Hz) and absence of an applied magnetic field using the dual Helmholtz/spectrophotometer system. Enzyme activity is assayed by measuring 4-nitrophenol formation (abs. 405 nm) with time. *Inset*. Shows a plot of the applied field (**AF**) time course minus the no applied field (**C**) time course. Conditions: [substrate] = 0.07 mM. **B**. Alkaline phosphatase activity in the presence (2.5 mT/75 Hz) and absence of an applied magnetic field using the Biostim system. *Inset*. Shows a plot of the applied field (biostim on, **bio**_**on**_) time course minus the no applied field (biostim off, **bio**_**off**_) time course. Conditions: [substrate] = 0.07 mM.

Fig 1**A** shows the time courses for the release of 4-nitrophenol from 4-nitrophenlyphosphate catalysed by alkaline phosphatase, in the absence and presence of a magnetic field (2.5 mT/75 Hz) applied using the dual Helmholtz/spectrophotometer system. The *inset* to Fig 1**A** shows a plot of the time course with applied field (**AF**) minus that in the absence of the applied field (**C**). Fig 1**B** shows similar time courses in the presence of the field applied by the Biostim apparatus. The difference between Biostim on (**bio**_**on**_) and Biostim off (**bio**_**off**_) is also shown, see *inset*. The activity of alkaline phosphatase (**AF**-**C**) was studied at five different substrate concentrations (0.07, 0.1, 0.25, 1, and 5 mM) and at each concentration experiments were carried out three times (5x3 = 15 experiments in total). Table 2 shows the mean difference in activity with standard deviation between the two field conditions. The activity of alkaline phosphatase (**bio**_**on**_-**bio**_**off**_) was also studied at one substrate concentration (0.07 mM), in triplicate. The mean and standard deviation of the difference in activity, (absorbance/min) between the “field on” and “field off” conditions were calculated to be 3x10^-5^ and 2x10^-5^ ΔA_405_/min respectively. This experiment using the Biostim has been designed to be as closely similar in methodology as possible to that carried out by Morelli *et al*. [**17**] in which they observed a marked decrease in enzyme activity.

**Table 2:** For alkaline phosphatase using the dual Helmholtz coil/spectrometer system, the mean (mean) value of the slopes fitted for AF-C for each substrate concentration is given. The mean value for each substrate concentration used was calculated from 3 separate experiments (n = 3), the slope for each experiment **AF-C** was fitted using a straight line of best fit with R^2^ values >0.98 in all cases. The standard deviation (**sd**) is also given. Similar (less extensive) data is also given for the alkaline phosphatase using the Biostim system (see results).

**[substrate] mM**	0.07	0.10	0.25	1.00	5.00
**mean (x10**^**-5**^**)**	-0.33	1.00	0.33	6.00	-1.67
**sd (x10**^**-5**^**)**	8.33	7.00	6.11	3.46	8.14

**Fig 2:**
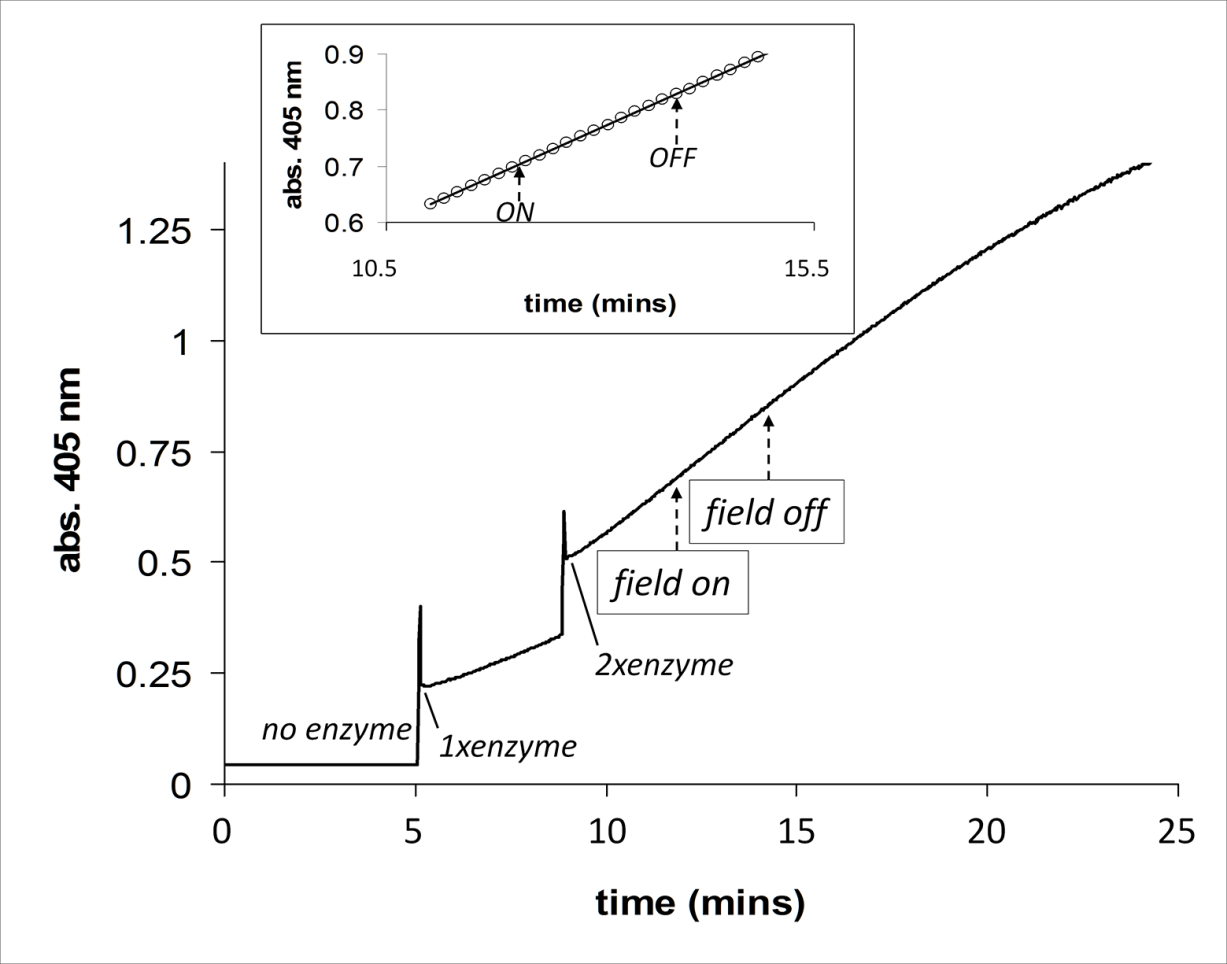
Alkaline phosphatase activity in the presence of an applied magnetic field (2.5 mT/75 Hz, field on/field off as indicated by arrows) using the dual Helmholtz/spectrophotometer system. At the start of the assay no enzyme is present in the reaction mixture. At time = ~5 mins an aliquot of enzyme/microsomes is added (denoted 1x enzyme), and at time = ~9 mins another aliquot of enzyme/microsomes is added (denoted 2x enzyme). *Inset*. A very good straight line fit of the data points for the region 1 min either side of where the field was applied in an on/off manner (field applied at 12 mins and switched off at 14 mins).

In Fig 2 the activity of alkaline phosphatase in the presence and absence (“on” and “off”) of an applied magnetic field is shown. At the start of the assay all reactant mixtures were placed in the cuvette which was positioned in the monitoring beam. At t = ~5 mins an aliquot of enzyme/microsomes was added (denoted 1x enzyme). It can be clearly seen that prior to this first addition of enzyme, no reaction was observed. A further aliquot of enzyme (2x total) was added at time = ~9 mins, and on this addition it can be seen that the rate of product formation doubles. The *inset* to Fig 2 shows an expanded region of the linear time course of the reaction in which the field (2.5 mT/75 Hz) was turned “on” and then “off”. A straight line of best fit is shown plotted through all the data points that start one minute before the field was applied to one minute after the field was switched “off” (R^2^ = >0.99), and this shows there is no change in slope during the time the field was applied. Further more detailed analysis was carried out on these experiments, where in a single experiment straight lines were fitted to the data points one minute before the field was turned “on” to one minute after the field remained “on” and, one minute after the field remained “on” to one minute after the field was turned “off”. This analysis revealed that there was no change in the slopes on the field being turned either “on” or “off” (R^2^ values = > 0.99). These experiments were carried out five times in total, and all the lines of best fit were analysed to ascertain if there was any deflection from linearity due to the field. No significant effect of the field was observed in any of the experiments.

**Fig 3:**
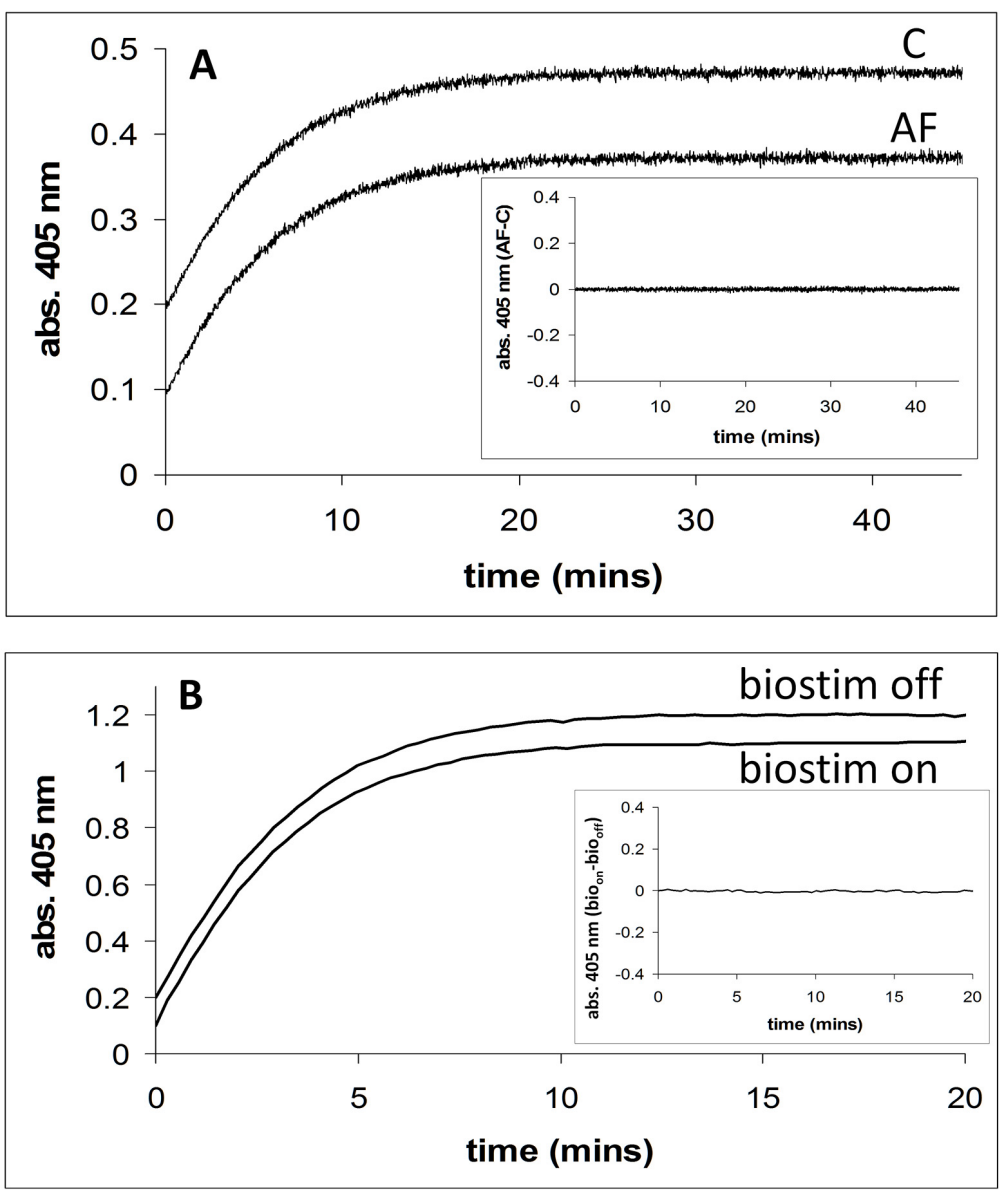
Acetylcholinesterase activity in the presence and absence of applied magnetic fields. **A**. Acetylcholinesterase activity in the presence (2.5 mT/75 Hz) and absence of an applied magnetic field using the dual Helmholtz/spectrophotometer system. Enzyme activity is assayed by measuring thionitrobenzoate formation (abs. 405 nm) with time. *Inset*. Shows a plot of the applied field (**AF**) time course minus the no applied field (**C**) time course. Conditions: [substrate] = 0.025 mM. **B**. Acetylcholinesterase activity in the presence (2.5 mT/75 Hz) and absence of an applied magnetic field using the Biostim system. *Inset*. Shows a plot of the applied field (biostim on, **bio**_**on**_) time course minus the no applied field (biostim off, **bio**_**off**_) time course. Conditions: [substrate] = 0.10 mM.

In Fig 3**A**, the activity (thionitrobenzoate formation, abs. 405 nm) of the enzyme acetylcholinesterase in the presence of an externally applied magnetic field (2.5 mT/75 Hz) is shown. The same reaction is also shown where no external field was applied. The *inset* to Fig 3**A** shows a plot of the applied field (**AF**) time course minus the no applied field (**C**) time course. Fig 3**B** shows the activity of acetylcholinesterase in the presence and absence of an externally applied magnetic field using the Biostim apparatus. The Biostim on (**bio**_**on**_) minus Biostim off (**bio**_**off**_) plot is also shown for this experiment (see *inset*). Using the dual Helmholtz/spectrophotometer system, the activity of acetylcholinesterase (**AF**-**C**) was studied at five different substrate concentrations (0.025, 0.05, 0.1, 0.2, and 0.5 mM) and at each concentration experiments were carried in triplicate (5x3 = 15 experiments in total). Table 3 shows the results of these experiments, with the mean and standard deviation for the difference in activity given. Using the Biostim apparatus and spectrometer system, the activity of acetylcholinesterase (**bio**_**on**_-**bio**_**off**_) was studied, in triplicate at each substrate concentration, at the same concentrations as for the dual Helmholtz coil/spectrometer system (5x3 = 15 experiments in total). These results are reported in Table 4. This experiment using the Biostim has been designed to be as closely similar in methodology as possible to that carried out by Morelli *et al*. [**17**] in which they observed a marked decrease in enzyme activity.

**Table 3:** For acetylcholinesterase using the dual Helmholtz coil/spectrometer system, the mean (mean) value of the slopes fitted for AF-C for each substrate concentration is given. The mean value for each substrate concentration used was calculated from 3 separate experiments (n = 3), the slope for each experiment **AF-C** was fitted using a straight line of best fit with R^2^ values >0.98 in all cases. The standard deviation (**sd**) is also given.

**[substrate] mM**	0.025	0.05	0.10	0.20	0.40
**mean (x10**^**-5**^**)**	0.00	-2.00	0.67	-1.00	3.00
**sd (x10**^**-5**^**)**	2.65	4.36	4.16	2.65	5.57

**Table 4:** For acetylcholinesterase using the dual Helmholtz coil/spectrometer system, the mean (mean) value of the slopes fitted for bio_on_-bio_off_ for each substrate concentration is given. The mean value for each substrate concentration used was calculated from 3 separate experiments (n = 3), the slope for each experiment **bio**_**on**_-**bio**_**off**_ was fitted using a straight line of best fit with R^2^ values >0.98 in all cases. The standard deviation (**sd**) is also given.

**[substrate] mM**	0.025	0.05	0.10	0.20	0.40
**mean (x10**^**-5**^**)**	-1.33	1.67	-13.0	4.00	2.67
**sd (x10**^**-5**^**)**	5.51	5.13	7.37	5.00	7.51

**Fig 4:**
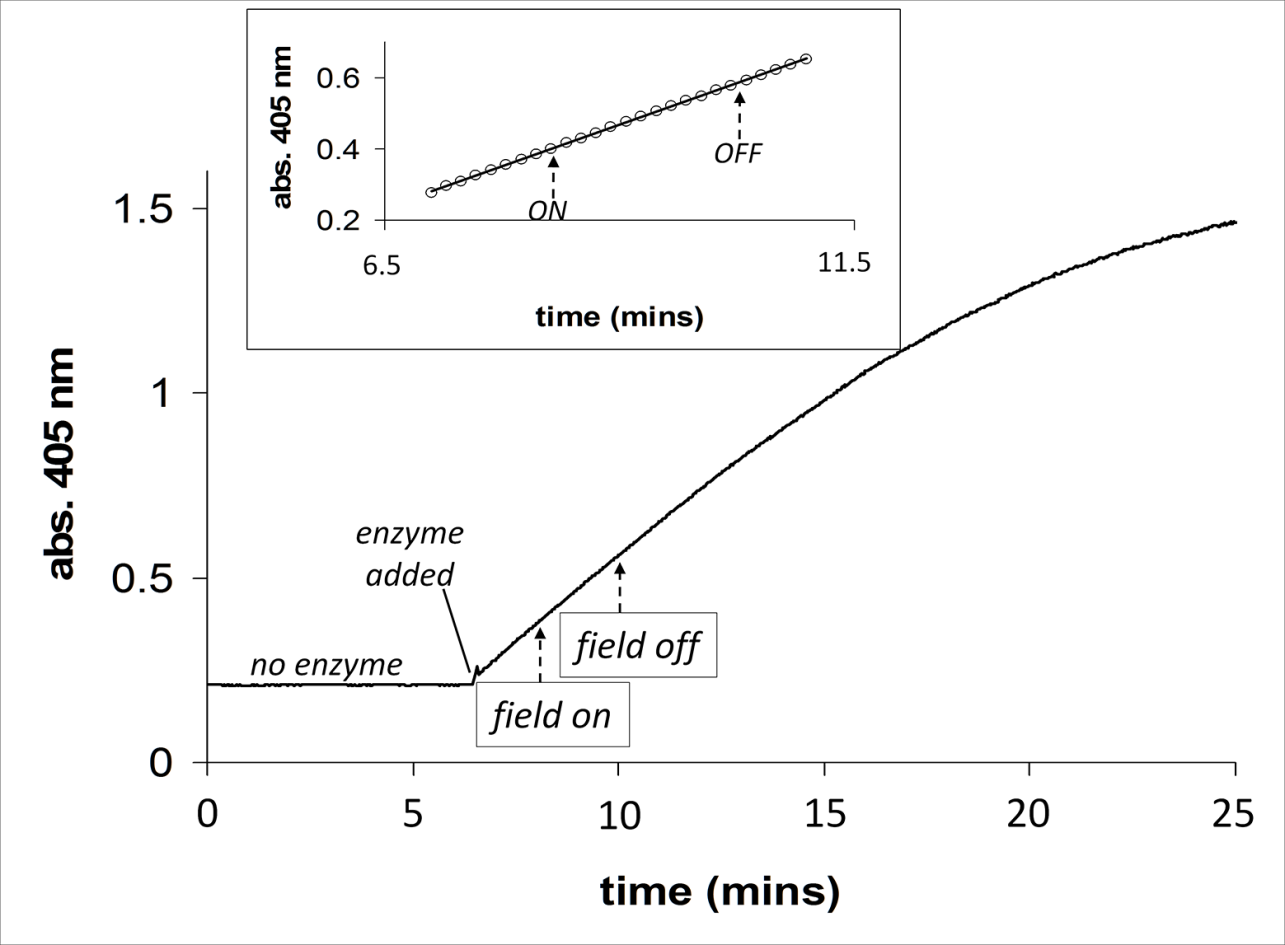
Acetylcholinesterase activity in the presence of an applied magnetic field (2.5 mT/75 Hz, field on/field off as indicated by arrows) using the dual Helmholtz/spectrophotometer system. At the start of the assay no enzyme is present in the reaction mixture. At time = ~6.5 mins an aliquot of enzyme/ghost cell membranes is added. *Inset*. A very good straight line fit of the data points for the region 1 min either side of where the field was applied in an on/off manner (field applied at 8 mins and switched off at 10 mins).

In Fig 4, the activity of acetylcholinesterase is shown in the presence and absence of an applied magnetic field on the same sample, the field being switched “on” and the “off” during the time course (indicated by arrows). At the start of this assay, the reaction mixture was placed in the cuvette, positioned in the monitoring beam, and at t = ~7 mins an aliquot of enzyme/ghost cell membranes was added. Prior to this first addition of enzyme no reaction was observed. The *inset* to Fig 4 shows an expanded region of the time course of the reaction where this was linear and the field (2.5 mT/75 Hz) was turned “on” and then “off”. A straight line of best fit is shown plotted through all the data points that start one minute before the field was applied to one minute after the field was switched “off” (R^2^ = >0.99), and this shows there is no change in slope during the time the field was applied. Further more detailed analysis was carried out on these experiments, where in a single experiment straight lines were fitted to the data points one minute before the field was turned “on” to one minute after the field remained “on” and, one minute after the field remained “on” to one minute after the field was turned “off”. This analysis revealed that there was no change in the slopes on the field being turned either “on” or “off” (R^2^ values = > 0.99). These experiments were carried out five times in total, and all the lines of best fit were analysed to ascertain if there was any deflection from linearity due to the field. No significant effect of the field was observed in any of the experiments.

**Fig 5:**
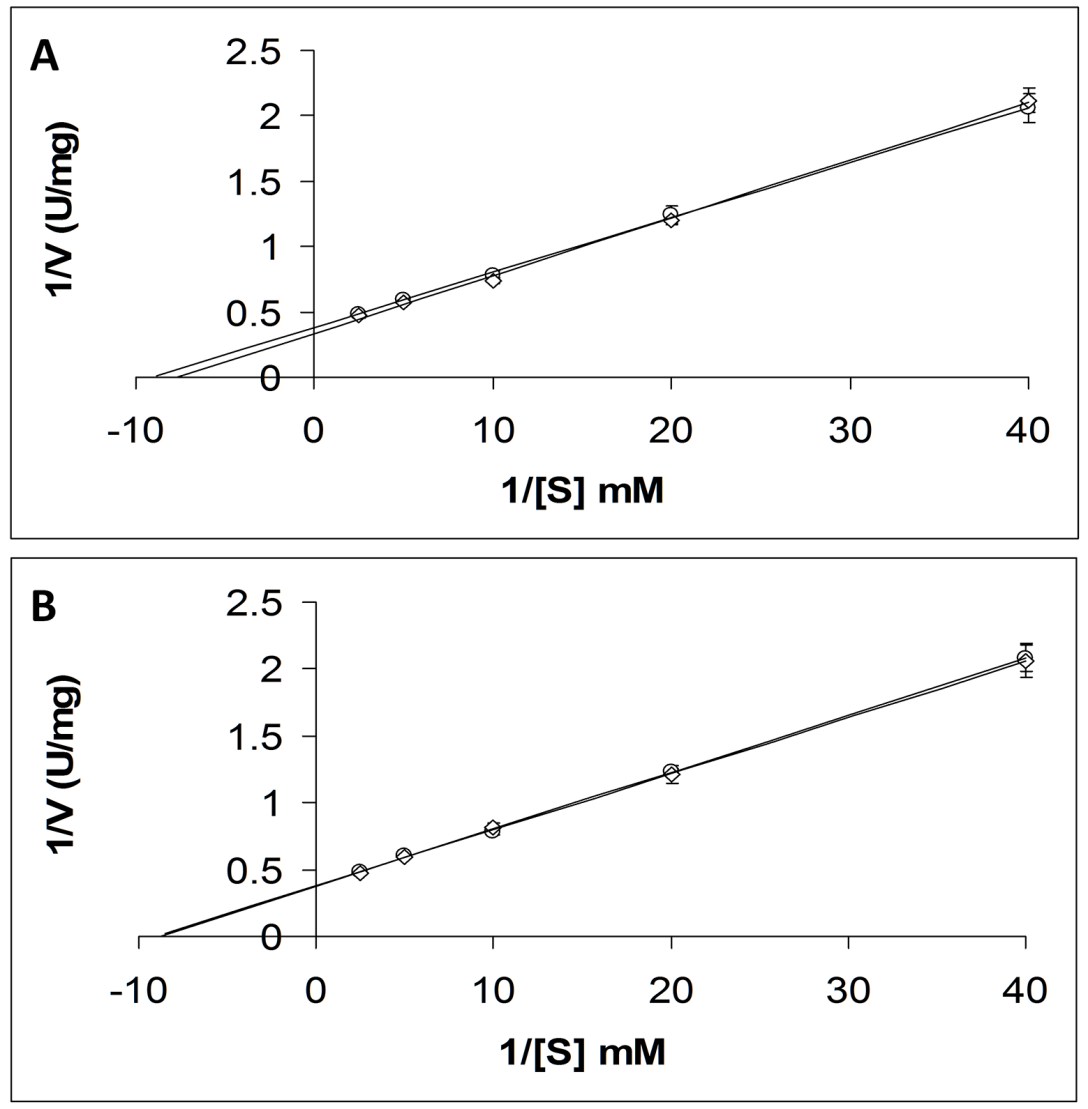
Lineweaver-Burk plots calculated from the Michaelis-Menten kinetics of acetylcholinesterase from ghost cell membranes. **A**. Plots for acetylcholinesterase activity in the presence and absence of an applied magnetic field using the dual Helmholtz/spectrometer system (2.5 mT/75 Hz). **B**. Plots for acetylcholinesterase activity using the Biostim apparatus. For each experiment using either apparatus, at any given substrate concentration, one sample was placed in the counter wound coil where no field could be applied and the other in the coil or Biostim where a field could be applied, and reactions were followed simultaneously. Data obtained in the absence of a field is denoted by **circles** and those in the presence of field by **diamonds**. The enzymatic activity V was measured in U/mg, which is defined as nmoles of substrate converted per minute per mg of protein (nmoles/minute/mg). The protein concentration in mg/ml was calculated using the Biuret method [**19**]), and these calculated values were used to establish the total protein concentration present in the assays.

In Fig 5**A** and 5**B**, Lineweaver-Burk plots have been calculated from the Michaelis-Menten kinetics displayed by acetylcholinesterase activity of ghost cell membranes. Fig 5**A** shows these plots obtained using the dual Helmholtz coil/spectrometer system and Fig 5**B** those for the Biostim spectrometer system. The data points shown for both plots are the mean values of three experiments at each substrate concentrations and the error bars are the standard deviation. In Table 5 the V_max_ and K_M_ values in the presence and absence of externally applied magnetic fields are given. The values are very similar to those reported by Morrelli *et al*. in the absence of field but in our experiments the V_max_ did not exhibit an approximately 50% diminution in the presence of a field. The analysis/presentation of this experiment using the Biostim, is almost identical to that carried out by Morelli *et al*. [**17**].

**Table 5:** For acetylcholinestersase the V_max_ and K_M_ values in the presence and absence of externally applied magnetic fields for both apparatus systems are calculated and given. The control coil in the dual Helmholtz system had no applied field administered in all experiments.

	Helmholtz	coils	Helmholtz	coils	Biostim	
	*field coil (no applied field)*	*control coil*	*field coil (2*.*5 mT/75Hz)*	*control coil*	*ON*	*OFF*
**K**_**M**_ **(mM)**	0.121	0.116	0.130	0.111	0.109	0.114
**V**_**max**_ **(U/mg)**	2.917	2.791	2.985	2.632	2.618	2.667

**Fig 6:**
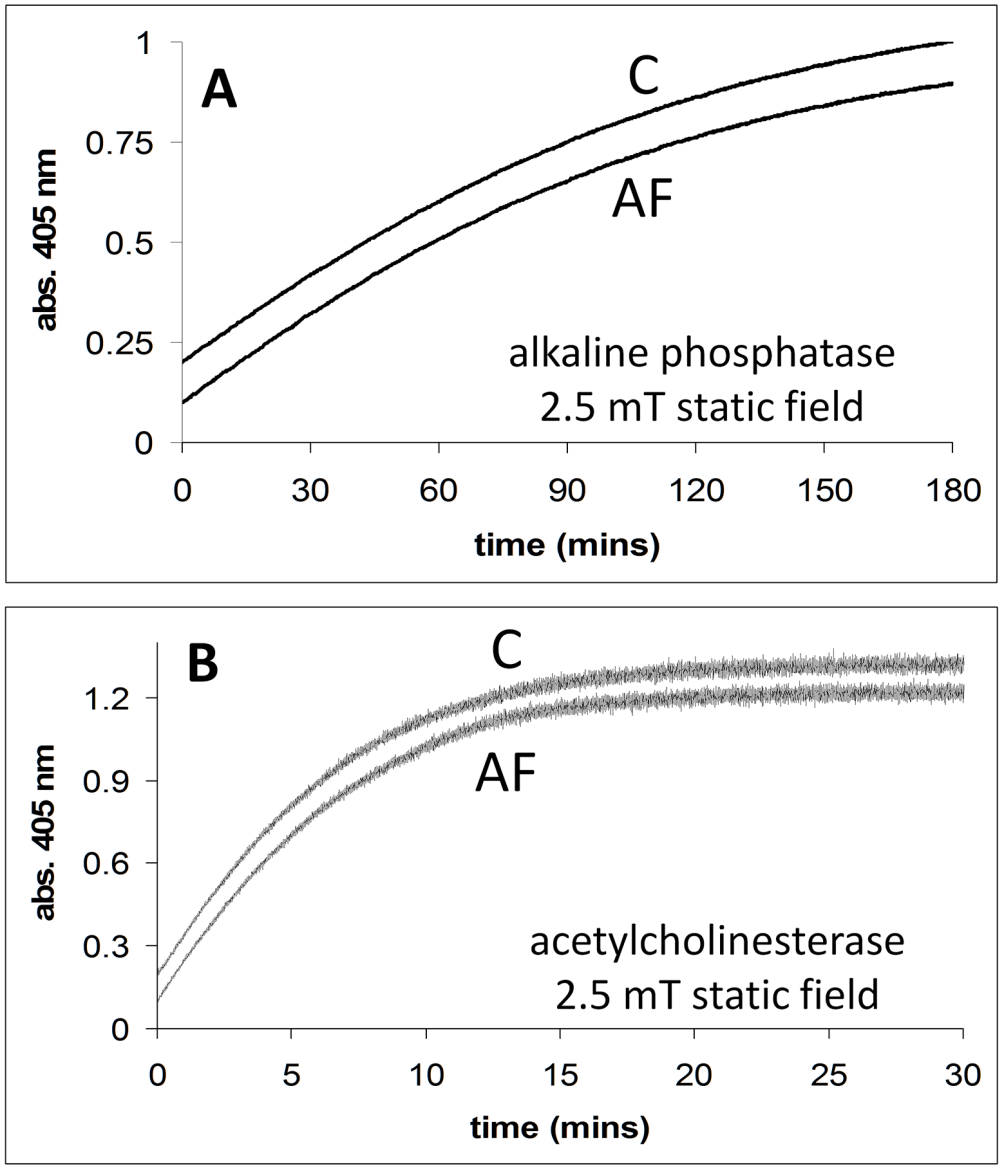
**A**. Alkaline phosphatase activity in the presence (2.5 mT static field) and absence of applied magnetic fields. Conditions: [substrate] = 0.07 mM. **B**. Acetylcholinesterase activity in the presence (2.5 mT static field) and absence of an applied magnetic field. Conditions: [substrate] = 0.025 mM. All reactions were carried out in the dual Helmholtz/spectrophotometer system. The applied field (**AF**) and no applied field (**C**) time courses are shown.

In Fig 6**A** and 6**B**, the activities of alkaline phosphatase and acetylcholinesterase are shown in the absence and presence of a 2.5 mT static field. In Fig 6**A** the time courses for the release of 4-nitrophenol from 4-nitrophenlyphosphate catalysed by alkaline phosphatase is shown, in the absence and presence of a magnetic field (2.5 mT static field) applied using the dual Helmholtz/spectrophotometer system. In Fig 6**B** the activity (thionitrobenzoate formation, abs. 405 nm) of the enzyme acetylcholinesterase in the absence and presence of an externally applied magnetic field (2.5 mT static field) is shown. We do not discuss these results, but show them for a more complete data set.

## Discussion

In contrast to the reports by Morelli *et al*. [**17**], we have been unable to detect magnetic field effects on the steady state kinetics of either acetylcholinesterase or alkaline phosphatase. This discrepancy cannot be explained in terms of the assay conditions as we have paid particular attention to reproducing the conditions used by these authors, although Morelli *et al*. [**17**] do not report the temperature at which their experiments were performed. We also followed closely the protocols that were reported by Morelli *et al*. for the preparation of the membrane bound enzymes alkaline phosphatase and acetylcholinesterase. In addition, to be sure that there were no differences in the way in which the field was applied, we undertook experiments both with the Helmholtz coil system and with the Biostim apparatus used in the earlier studies.

One clear difference in which our method of assay is distinct from the previous investigators is in regard to the method used to monitor activity, the earlier reports employing fixed-time assays while we have used real-time spectral assays. It is difficult to see how this methodological difference can account for the discrepancy between the studies.

The mechanism proposed by Morelli *et al*. to account for the magnetic field effect is that the activities of the enzymes depend upon the fluidity of the phospholipid membrane in which they are embedded. The field, acting through the diamagnetic susceptibility of the membrane, is suggested to orient the lipid and restrain its thermal motion and thus affect the enzyme’s activities. In support of this proposal Morelli *et al*. show that in the presence of Triton x-100, a detergent that dissolves the membrane, the effect of the field is abolished. Although this explanation is feasible in principle the fields that were used in the investigation make this unlikely on energetic grounds. Given a field of ~10 mT the low magnetic susceptibility for diamagnetic materials such as lipid molecules indicate an aligning energy of ~10^−27^ J. This may be compared with thermal energy at 300°K (~kT, where k is the Boltzmann constant) of ~10^−21^ J, i.e. a factor of 10^6^ fold greater. Thus one may presume that fields of ~1 mT or less will have undetectable influence on membrane fluidity (see for example [**5**]). Studies that have demonstrated the effects of magnetic fields on phospholipids membranes have typically used field of the order of 1 T or greater [**23**]. Water soluble proteins or protein domains tethered to membranes can be affected by magnetic fields through the field-induced orientation/phase change in the associated membrane e.g. bacterial purple membrane, but again at high field *~*10 T [**24**]. Effects at lower field strengths have, however, been reported in membranes containing ion channels. For example miniature endplate potentials recorded in the isolated murine presynaptic neuromuscular junction were inhibited at relatively low field and exhibited an absolute flux density threshold of 39.7 mT [**25**] The authors postulate that this effect is mediated through reorientation of diamagnetic molecular domains within the membrane. This behaviour has some similarity to that reported by Ravera *et al*. [**18**], and one may surmise that if the synaptosomal membrane associated acetylcholinesterase could also be associated with ion channels within the membrane that reorientation of these may influence the esterase activity.

Alternatively, small changes in temperature, especially in the region of a phase transition, may alter membrane fluidity and induce measurable effects in enzyme activity [**26**]. The field effects reported by Morelli *et al*. for alkaline phosphatase exhibits a remarkably sharp transition between 130 μT and 150 μT the activity halving over this 20 μT range. It is difficult to explain such behaviour of system in response to a magnetic field without recourse to a mechanism involving correlated radical pairs. It is more reminiscent of phase transition that is coupled to temperature, for example the phase transition found in phospholipids lipid membrane that leads to enhanced fluidity [**26**]. The temperature dependence of acetycholinesterase, at least, is complex with clear inflection in the Arhenius plot at around 30°C. indicating a sharp change in activation energy. Furthermore if enzymes are not assayed at saturating substrate concentration an increase in temperature will not only increase the rate (and hence V_max_) it is also likely to alter K_M_ and lead to a change in observed velocity which may be positive or negative.

During the course of our investigations we became aware of the need for extremely close control of temperature and continuous monitoring of the temperature of sample and reference reaction mixtures. In our hands differences between sample and reference of <0.1°C (at 25°C) lead to measurable differences in these membrane bound enzyme’s activities. We suggest, therefore, that the complex and confounding influences off temperature on these complex physical systems, comprising mixed phospholipid membrane in which is embedded a dynamic protein possessing catalytic activities involving multi-step mechanisms, may account for why Morelli *et al*. observed magnetic field effects while we did not.

Although we have attempted to reproduce closely the experimental protocols and experimental conditions of Morrelli and Ravera there may be some important differences of which we are unaware. For example we do not know the phase transition temperature of the membranes we used. One may suppose that any magnetic effect mediated by a reorientation of lipid or protein within the membrane must depend strongly on temperature. Such orientation would be energetically easier close to the membrane transition temperature and more difficult below this temperature. If for example the lipid compositions of the membranes were different between the preparations we used and those the group based in Italy used, a not unreasonable proposition given the influence of nutritional differences on membrane composition, then a temperature of 25°C may or close or relatively far from the membrane transition temperature. This being the case parallel experiments at this temperature may yield conflicting results.

## References

[1] Kirschvink JL, Kobayashi-Kirschvink A, Diaz-Ricci JC, Kirschvink SJ. Magnetite in human tissues: a mechanism for the biological effects of weak ELF magnetic fields. Bioelectromagnetics Supplement 1 1992: 101–113. 1285705 10.1002/bem.2250130710

[2] Barnes F. Mechanisms for electric and magnetic field effects on biological cells. IEEE Trans. Magn. 2005;11: 4219–4224.

[3] Havas M. Biological Effects of Low Frequency Electromagnetic Fields In: Clements-Croome D (Ed.). Electromagnetic Environments and Health in Buildings. Spon Press, London 2004: 535.

[4] Labes MM. 10.1038/211968a0 Letts Nature. 1966;11(968). 5968306

[5] Rosen AD. Studies on the Effect of Static Magnetic Fields on Biological Systems. PIERS ONLINE. 2010;6(2): 133–136.

[6] Ritz T, Adem S, Schulten K. A model for photoreceptor-based magnetoreception in birds. Biophysical Journal. 2000;78: 707–718. 10653784 10.1016/S0006-3495(00)76629-X PMC1300674

[7] Schulten K, Swenberg CE, Weller A. A biomagnetic sensory mechanism based on magnetic field modulated coherent electron spin motion. Zeitschrift fur Physikalische Chemie. 1978;111: 1–5.

[8] Ball P. Physics of life: The dawn of quantum biology. Nature. 2011 June 15. 10.1038/474272a 2011;474: 272–274. 21677723

[9] Wiltschko W, Wiltschko R. Magnetic Orientation in Birds. The Journal of Experimental Biology. 1996;199: 29–38. 9317275 10.1242/jeb.199.1.29

[10] Kobayashi A, Kirschvink J. Magnetoreception and Electromagnetic Field Effects: Sensory Perception of the Geomagnetic Field in Animals and Humans. Magnetoreception and EMF Effects. 1995;21: 367–394.

[11] Rogers CT, Hore PJ. Chemical magnetoreception in birds: The radical pair mechanism. PNAS. 2008;106(2): 353–360. 10.1073/pnas.0711968106 PMC2626707 19129499

[12] Solov’yov IA, Schulten K. Reaction kinetics and mechanism of magnetic field effects in cryptochrome. Journal of Physical Chemistry B. 2012;116: 1089–1099. 10.1021/jp209508y PMC3266978 22171949

[13] Solov’yov IA, Domratcheva T, Shahi ARM, Schulten K. JACS. 2012;134: 8046–1852. 10.1021/ja3074819 PMC3500783 23009093

[14] Muller P, Ahmad M. Light-activated cryptochrome reacts with molecular oxygen to form a flavin-superoxide radical pair consistent with magnetoreception. JBC. 2011;286: 21033–21040. 10.1074/jbc.M111.228940 PMC3122164 21467031

[15] Buchachenko AL, Kusnetsov DA. Magnetic field affects enzymatic ATP synthesis. J. Am. Chem. Soc. 2008;130: 12868–12869. 10.1021/ja804819k 18774801

[16] Crotty D, Silkstone G, Poddar S, Ranson R, Prina-Mello A, Wilson MT, Coey JMD. Reexamination of magnetic isotope and field effects on adenosine triphosphate production by creatine kinase. PNAS. 2012;109(5): 1437–1442. 10.1073/pnas.1117840108 22198842 PMC3277194

[17] Morelli A, Ravera S, Panfoli I, Pepe IM. Effects of extremely low frequency electromagnetic fields on membrane-associated enzymes. Archives of Biochemistry and Biophysics. 2005;441: 191–198. 16126157 10.1016/j.abb.2005.07.011

[18] Ravera S., Bianco B., Cugnoli C., Panfoli I., Calzia D., Morelli A., Pepe I. M.. Sinusoidal ELF magnetic fields affect acetylcholinesterase activity in cerebellum synaptosomal membranes. Bioelectromagnetics. 2010;31(4): 270–276. 10.1002/bem.20563 20041436

[19] Caseli L, Oliveira RG, Masui DCM, Furriel RP, Leone FA, Maggio B, Zaniquelli MED. Influence of a stationary magnetic field on acetylcholinesterase in murine bone marrow cells. Langmuir. 2005;21: 4090–4095. 15835979

[20] Stegemann S, Altman KI, Muhlensiepen H, Feinendegen LE. Influence of a stationary magnetic field on acetylcholinesterase in murine bone marrow cells. Radiat. Environ. Biophys. 1993;1: 65–72. 10.1007/BF01213132 8460216

[21] Hamdy HA, Mahmoud BF, Shalaby TE, El-Sharkawy AM, Farag IMF. Effect of static and alternating magnetic fields on acetylcholinesterase and monoamine oxidase activities in the brain of mice. J. Invest. Biochem. 2013. 10.5455/jib.20130716075920 2013;3(4): 133–137.

[22] Fenk CJ, Kaufman N, Gerbig DG. Protein detection and measurement. J. Chem. Educ. 2007;84: 1676–1678.

[23] Orchard AF. Magnetochemistry (Publisher: OUP Oxford, 2003, ISBN 9780198792789).

[24] Polk C, Postow E. Handbook of Biological effects of electromagnetic field, Second Edition, CRC Press 1996 Chapter 3 “Biological effects of static magnetic fields” by Frankel RB and Liburdy RP: 159–162.

[25] Rosen D. Studies on the Effect of Static Magnetic Fields on Biological Systems. BBA (Biomembranes). 1994;1193: 62–66.8038195

[26] Gaffney BJ, McConnell HM. Effect of a Magnetic Field on Phospholipid Membranes. Chemical Physics Letters. 1974;24: 310–313.

